# Sex Disparities in PAD Patients: Retrospective Study Utilizing MIMIC-IV v3.1 Database

**DOI:** 10.3390/jcm14103304

**Published:** 2025-05-09

**Authors:** Sanaullah Mojaddedi, Geran Maule, Javairia Jamil, John Rickards, Margaret K. Ohama, Mohammad Khraisat, Abdallah Rayyan, Suzanne Zentko

**Affiliations:** 1 Graduate Medical Education, University of Central Florida College of Medicine, Orlando, FL 32827, USA; geran.maule@hcahealthcare.com (G.M.);; 2 Internal Medicine Residency Program, HCA Florida North Florida Hospital, Gainesville, FL 32605, USA; 3 College of Medicine, Gulf Medical University, Ajman P.O. Box 4184, United Arab Emirates; 4 Department of Internal Medicine, Mercer University School of Medicine, Macon, GA 31207, USA; john.rickards@atriumhealth.org; 5 The Cardiac and Vascular Institute, Gainesville, FL 32605, USA; mkohama@tcavi.com

**Keywords:** peripheral artery disease, gender disparities, atherosclerosis, endovascular interventions, epidemiology, health outcomes

## Abstract

**Background**: Peripheral artery disease (PAD) is a progressive atherosclerotic condition associated with significant morbidity and mortality. While PAD prevalence is comparable between sexes, women tend to have worse clinical outcomes, higher rates of disability, and are underdiagnosed and undertreated compared to men. This study examines sex differences in PAD presentation, diagnosis, and treatment outcomes using the Medical Information Mart for Intensive Care (MIMIC)-IV v3.1 database. **Methods**: A retrospective cohort study was conducted using electronic health records from the MIMIC-IV v3.1 database, identifying patients diagnosed with PAD between 2008 and 2022. Patient selection was based on International Classification of Diseases (ICD)-9 and ICD-10 codes. The following two datasets were constructed: an admission-level dataset (6468 admissions, 3913 unique patients) and a patient-level dataset aggregating multiple admissions per individual. Key variables included demographics, hospitalization details, procedure rates, and clinical outcomes. Sex-based comparisons were performed to assess disparities in disease burden, intervention rates, and mortality. **Results**: The study cohort comprised 3913 PAD patients. Women were significantly older than men at time of admission (mean 70.78 vs. 68.97 years, *p* < 0.05) and had lower rates of procedural intervention across all categories, including angioplasty (12.85% vs. 15.39%) and bypass grafting (14.74% vs. 16.98%). Despite similar Intensive Care Unit (ICU) admission rates (30.56% in females vs. 31.73% in males), women experienced greater delays in PAD diagnosis and treatment initiation. The in-hospital mortality rate was comparable between sexes (6.62% vs. 6.92%). Women presented more frequently with atypical or asymptomatic PAD, leading to delays in diagnosis and specialist referrals. **Conclusions**: This study highlights significant sex disparities in PAD diagnosis and management. Women with PAD are older at diagnosis, receive fewer procedural interventions, and experience delayed clinical recognition, contributing to a higher cumulative disease burden. These findings underscore the need for sex-specific diagnostic criteria, improved clinical awareness, and equitable treatment strategies to optimize PAD outcomes in women.

## 1. Introduction

Peripheral artery disease (PAD) is a progressive atherosclerotic condition affecting over 200 million individuals worldwide. It is associated with significant impacts on morbidity, functional impairment, and mortality. It is characterized by arterial narrowing and a reduced blood flow, primarily in the lower extremities but also in the upper extremities, leading to ischemia, claudication, and limb-threatening complications [1].

Although historically considered more prevalent in men, emerging evidence indicates a comparable or higher PAD prevalence in women, particularly among older populations [2,3]. Despite a similar disease burden, women continue to experience worse clinical outcomes, delayed diagnoses, and undertreatment. These sex differences are multifactorial, involving biological, clinical, and societal factors [1,2,4,5]. Women with PAD frequently present with atypical symptoms or are asymptomatic, leading to underdiagnosis and delayed treatment [1,5]. Furthermore, anatomical and physiological differences—such as smaller vessel diameters, more diffuse atherosclerotic disease, and increased arterial stiffness—make women less likely to be considered candidates for revascularization procedures [6,7,8].

Access to healthcare services further contributes to sex differences in PAD diagnosis, treatment, and outcomes [4]. In 2012, the American Heart Association made a call for action to address sex-related disparities in PAD, but at the time of writing, we lack studies addressing this issue [9].

In this study, we utilized the Medical Information Mart for Intensive Care (MIMIC)-IV v3.1 [10], a large, de-identified electronic health record database, to examine sex-based differences in PAD presentation, procedural interventions, and outcomes among hospitalized patients. Utilizing International Classification of Diseases (ICD)-9 and ICD-10 codes, we identified patients diagnosed with PAD between 2008 and 2022 and assessed their hospitalization details, procedural interventions, and clinical outcomes. By analyzing both admission-level and patient-level datasets, we aimed to evaluate procedure utilization, disparities in treatment patterns, and mortality across sexes.

## 2. Background

Several factors are believed to influence sex disparities among PAD patients, including socioeconomic factors, pain perception, plaque characteristics, clinician recognition, and diagnostic limitations.

### 2.1. Sociocultural and Economic Barriers

Women may experience greater barriers to healthcare access, including a lower socioeconomic status and reduced healthcare literacy on a global scale [4]. Women may have fewer financial resources to see physicians, leading to worse outcomes and exposing sex disparities [4]. A study using the National Inpatient Sample Database examined patients diagnosed with critical limb ischemia between 2005 and 2014 and concluded that less comprehensive insurance coverage, such as Medicaid, resulted in more amputations [11]. Lastly, clinician diversity may be another significant obstacle, as sex discordance between the clinician and the patient can negatively impact the patient–physician relationship, leading to worse outcomes [4]. In the United Kingdom, a national report from 2020 revealed that 35% of all surgical trainees and 14% of vascular consultants were females [12]. These disparities create additional barriers for women with PAD.

### 2.2. Pain Perception

Another factor that delays diagnosis and treatment is an atypical presentation of pain. Less than one-third of patients have typical intermittent claudication, while the majority suffer from atypical symptoms, (leg pain with rest, pain that does not improve within ten minutes of exercise cessation, and burning pain) or are asymptomatic [13,14]. A systematic review and meta-analysis including 1,929,966 patients with PAD determined that women present less often with intermittent claudication compared to men (OR 0.78, 95% CI 0.72–0.84, *p* < 0.001), while rest pain is more prevalent in women (OR 1.40, 95% CI 1.22–1.60, *p* < 0.001) [5]. This evidence was of a low quality given the inconsistency and subjectivity of symptoms, and further studies are needed to further investigate these findings. Females have also been found to have a higher pain tolerance and can be more accepting of functional limitations [15]. The Women’s Health and Aging study, which included 847 disabled women aged 65 and older, revealed that only 14% of females with PAD had a history of intermittent claudication, while 64% were asymptomatic [16]. In a four-year study by McDermott et al. [17], men and women with PAD were assessed with a 6 min walk test and mobility disability. The researchers found that women were less likely to continuously complete the 6 min walk test and were more likely to develop mobility disability compared to their male counterparts (HR 2.3, 95% CI 1.3–1.1, *p* = 0.004 and HR 1.8, 95% CI 1.3–3.0, *p* = 0.03, respectively) [17]. Despite their lack of symptoms, females suffer from a rapid functional decline and poorer quality of life compared to males [17,18].

### 2.3. Plaque Characteristics

The ELSA-Brazil study (Brazilian Longitudinal Study of Adult Health) and a Finnish study both investigated the effects of carotid intima media thickness (IMT) and concluded that a higher IMT correlates with greater atherosclerotic disease [6,7]. The PROSPECT study (Providing Regional Observations to Study Predictors of Events in the Coronary Tree) included more than 600 patients from an atherosclerotic prevention clinic and revealed that women had a greater degree of stenosis than men [8]. Although these studies primarily focused on CAD, we can extrapolate similarities to PAD, as both diseases are essentially atherosclerosis [19]. Unfortunately, there is a lack of studies regarding sex differences among PAD plaque characteristics.

### 2.4. Diagnostic Limitations

The initial modality for PAD diagnosis is the Ankle–Brachial Index (ABI), a cost effective, non-invasive test with wide availability [1]. On average, men are taller than women, leading to a higher ankle systolic pressure, as arterial pressures increase with a greater distance from the heart, thereby the numerator for ABI tends to be higher in males [20,21]. In a cross-sectional analysis of 1775 healthy individuals in the Multi-Ethnic Study of Atherosclerosis (MESA) cohort, the ABI measurement was, on average, 0.02 units lower in women compared to men (−0.02, 95% CI −0.03–0.006), even when adjusting for height and confounders [21]. Although these sex differences are statistically significant, they are likely negligible in clinical practice, as ABI falls into the following four categories: normal 1.00–1.40, borderline 0.91–0.99, abnormal ≤ 0.90, and noncompressible > 1.40 [1].

### 2.5. Delayed Clinical Recognition and Referral

Healthcare providers may have a lower clinical suspicion for PAD in women, particularly in those who present with non-specific or atypical symptoms, often leading to misdiagnosis and treatment delays. Historically, PAD has been perceived as a “male disease”, which contributes to implicit provider bias in recognizing and evaluating PAD in women [22]. This often leads providers to attribute symptoms to musculoskeletal or other non-vascular causes. Notably, women are more frequently misdiagnosed with musculoskeletal disorders when they actually have PAD [23]. This bias results in fewer screenings, delayed referrals to specialists, and underuse of guideline-directed therapies for PAD in women [4]. Instead of referring women with suspected PAD to vascular specialists, primary care providers often opt for conservative management [4]. A study from Germany including over 17 million patients between 2009 and 2018 concluded that female patients with PAD were less likely to be referred to vascular surgery and angiography compared to their male counterparts, at 1.5% vs 3.2% and 1% vs 1.7%, *p* < 0.05, respectively [24]. Geographic disparities further widen this gap, with women in rural areas having less access to vascular specialists, therefore reducing their likelihood of timely referrals and treatment, increasing their risk for complications [25]. Due to these disparities in clinical recognition and referrals, women with PAD have more severe disease at presentation and tend to have worse outcomes [23].

### 2.6. Biological Mechanisms

Sex-based differences in PAD are partly driven by hormonal and genetic influences. Premenopausal women benefit from the vasoprotective properties of estrogen, which enhances nitric oxide bioavailability, antioxidant defenses, and anti-inflammatory pathways [26]. The abrupt decline in estrogen in postmenopausal women accelerates vascular aging and contributes to more severe and diffuse atherosclerotic disease [27,28]. Beyond hormonal regulation, X-linked genetic and epigenetic mechanisms also hold the possibility to independently modulate vascular responses. Additionally, pathologies predominantly seen in the female population, such as auto-immune disorders and preeclampsia, pre-dispose the female population to PAD [2]. These findings underscore the need for sex-specific screening and management strategies.

## 3. Materials and Methods

### 3.1. Data Source

This study utilized data from the MIMIC-IV v3.1 database [10], a publicly available, de-identified dataset containing electronic health records from patients admitted to Beth Israel Deaconess Medical Center (BIDMC) in Boston, MA, between 2008 and 2022. The dataset includes patients admitted through the Emergency Department (ED) and the Intensive Care Unit (ICU), providing detailed insights into diagnoses, procedures, vital signs, laboratory values, medication administration, and clinical outcomes.

MIMIC-IV is a retrospective cohort dataset and has been widely used for epidemiological and clinical research due to its high-quality structured data [10]. Access to the dataset was granted through the PhysioNet platform after completion of the required ethics training.

This study is a retrospective cohort analysis of patients with PAD identified in the MIMIC-IV v3.1 database. The cohort includes patients admitted to the ED or ICU with diagnosed PAD between 2008 and 2022, with a specific focus on procedural interventions and patient outcomes.

### 3.2. Patient Selection

Patients with PAD were identified using ICD-9 and ICD-10 diagnosis codes from the MIMIC database. The codes were selected based on their established use in classifying PAD, including atherosclerosis of native arteries and unspecified peripheral vascular disease. PAD-related ICD-9 and ICD-10 diagnosis and procedure codes were selected based on a combination of the following:

- Prior research on PAD classification.
- Expert consensus from vascular surgery and interventional radiology guidelines (ACC/AHA and SVS).
- Review of existing MIMIC-IV documentation.

### 3.3. Identification of Surgical Interventions

We examined the following six major procedures commonly performed in PAD patients:

- Amputation—including minor (toe and foot) and major (below-knee and above-knee) amputations.
- Atherectomy—a minimally invasive procedure that removes plaque from the arteries.
- Endarterectomy—the surgical removal of plaque from an artery.
- Angioplasty—the placement of a balloon to restore blood flow through an artery.
- Stent placement—the placement of a stent to restore artery patency.
- Bypass grafting—an autologous or synthetic graft used to bypass blood flow around an occluded artery.

A comprehensive list of ICD-9 and ICD-10 procedure codes corresponding to these interventions was compiled from the existing literature, reference manuals, and MIMIC-IV documentation. These codes were extracted from the hospital procedural records.

### 3.4. Data Processing and Dataset Construction

After identifying the relevant procedures, we constructed two datasets for analysis.

#### 3.4.1. Admission-Level Dataset (Peripheral Artery Disease _Patients)

- This dataset included all hospital admissions (not unique patients).
- Each patient was categorized based on whether they underwent any of the six interventions during their hospital stay.
- A binary indicator (1 = Yes and 0 = No) was assigned for each procedure.
- Total admissions in the dataset: 6468, representing 3913 unique patients, indicating that some patients had multiple hospitalizations.

The dataset also included key demographic and clinical variables, as follows:

- Demographics: age, sex, race, marital status, and primary language.
- Hospitalization details: admission/discharge times, length of stay (LOS), and in-hospital mortality.
- Insurance coverage: insurance type recorded at admission.
- ICU-specific data: whether the patient was admitted to intensive care and ICU LOS.

#### 3.4.2. Patient-Level Dataset (Peripheral Artery Disease_Patients_v2)

To examine long-term patient outcomes, we aggregated multiple admissions into a single row per patient, ensuring that each patient’s full procedural history was preserved.

Key Aggregation Methods:

- Procedural history: If a patient underwent a procedure during any admission, they were flagged as “1” in the patient-level dataset.
- Hospital and ICU LOS: Summed across all admissions to capture the total hospitalization burden.
- In-hospital mortality: If a patient died during any admission, they were marked as “1” (deceased) to prevent misclassification.
- Demographic variables: Sex, age, race, marital status, insurance type, and primary language were retained based on the most recent hospital admission.

Since some patients were admitted multiple times for PAD-related care, analyzing outcomes at the admission level could lead to redundant counting and bias in mortality and procedural rates. By aggregating admissions into a single patient-level dataset, we aimed to provide a clearer representation of cumulative disease burden and intervention history.

### 3.5. Exclusion Criteria and Handling of Missing Data

Patients were included if they had at least one hospital admission with a PAD diagnosis. No admissions were excluded based on missing demographic or procedural data, ensuring a complete capture of all PAD-related hospitalizations. Missing values for demographics were carried forward from the most recent admission to preserve consistency.

Primary and Secondary Outcomes:

- Primary outcomes:
  ○ In-hospital mortality (binary: 1 = deceased and 0 = survived).
  ○ ICU admission rate (binary: 1 = admitted to ICU and 0 = general ward admission).
- Secondary outcomes:
  ○ Hospital and ICU LOS (continuous).
  ○ Intervention rates (percentage of patients undergoing each PAD procedure).
  ○ Insurance and marital status distributions (to assess socioeconomic disparities).

By creating both an admission-based and a patient-level dataset, we enabled the following two complementary analyses:

1. Procedure frequency and trends per admission.
2. Long-term patient outcomes and cumulative procedural burden.

## 4. Results

This analysis included 3913 patients, with a mean age of 69.7 years (Table 1). The majority were white (2564) and male (2326). Medicare was the most common insurance type (2859). The median hospital length of stay (LOS) was 11.03 days, while the median ICU LOS was 4.27 days. Overall, 1223 patients (31.25%) required ICU admission, and the in-hospital mortality rate was 6.8%. Regarding procedures, bypass grafting was the most frequently performed (629 cases), followed by stent placement (577) and angioplasty (562).

This analysis compared 1587 female and 2326 male patients (Table 2). The mean age was slightly higher in females (70.78 years) compared to males (68.97 years). ICU admission rates were comparable between sexes (30.56% in females vs. 31.73% in males). The mean LOS was slightly longer in males (4.41 days) than females (4.05 days). Males also had a longer median hospital LOS (7.0 days vs. 6.0 days). In-hospital mortality rates were similar (6.62% in females, 6.92% in males). A higher number and greater raw percentage of males underwent procedures compared to females, consistent across all procedures (Table 3). Regarding marital status, a larger proportion of males were married, whereas more females were widowed. Medicare was the predominant insurance type for both sexes. Some key characteristics regarding patients above 60 can be found in Table 4 with similar in-hospital mortality among males vs. females (7.29% vs 7.45%, respectively). Table 5 shows the distribution of age groups categorized by sex.

## 5. Discussion

The findings of this study highlight significant sex disparities in the diagnosis, treatment, and outcomes of PAD, adding to the growing body of literature on sex-based differences in vascular disease. Despite an equivalent or higher prevalence of PAD in women, our cohort revealed that women were admitted for PAD at a later age, received fewer procedural interventions, and experienced a worse cumulative disease burden compared to men. These findings align with previous studies indicating that PAD in women is often underdiagnosed and undertreated, leading to increased morbidity and functional decline.

Notably, our study observed that women with PAD were significantly older at time of admission compared to men (70.78 vs. 68.97 years, *p* < 0.05). Similarly, a recent retrospective study by Martelli et al. conducted in Italy with 2399 chronic limb-threatening ischemia patients found that women were more likely to be above 75 years of age at time of admission compared to men (63.2% vs. 40.1%, *p* = 0.0001) [29]. This delay may be attributed to atypical symptom presentation, where women infrequently report classic intermittent claudication but likely present with rest pain or asymptomatic PAD. Prior studies have demonstrated that women tend to experience non-specific and atypical symptoms that do not immediately prompt vascular evaluation. These differences in symptomatology likely contribute to delayed recognition and subsequent treatment initiation, placing women at a higher risk of having advanced disease at presentation.

Additionally, we found that procedural intervention rates were lower in women across all major revascularization strategies, including bypass grafting, stent placement, and angioplasty. This disparity is multifactorial, with potential explanations including sex biases in clinical presentation patterns, differences in anatomical characteristics, and varying responses to interventions. Moreover, implicit biases in clinical decision making may lead to the under-recognition of PAD, with subsequent delays in treatment [4,23,24,25].

Despite these reported treatment disparities, our study found no statistically significant difference regarding in-hospital mortality rates between men and women (6.62% vs. 6.92%). While this may suggest that women do not experience worse short-term survival, it cannot extrapolate to long-term functional outcomes, post-discharge complications, or quality of life deterioration—factors that are particularly relevant in a population with high rates of limb-threatening ischemia. Women with PAD have been shown to experience faster functional decline and higher rates of disability compared to men, likely exacerbated by delayed diagnosis and undertreatment [5,17,18].

Several systemic factors contribute to the observed sex differences in PAD diagnosis and treatment, which include limitations of socioeconomic factors, pain perception, delayed clinical recognition, diagnostic limitations, healthcare access barriers, and the underrepresentation of women in clinical trials [4].

The implications of these findings are far-reaching. Given that PAD is a major cause of limb loss, cardiovascular events, and functional decline, addressing sex disparities in its diagnosis and treatment is critical to improving patient outcomes [1]. To address these disparities, we must first raise awareness so that clinicians can maintain a high index of suspicion for PAD in women with atypical presentation. Secondly, the development of sex-specific diagnostic criteria accounting for atypical PAD presentations is crucial to assist clinicians with diagnosis and management. Thirdly, we should implement strategies to reduce biases in vascular referral patterns, allowing for earlier revascularization procedures. Lastly, we should create equitable access to healthcare for women with socioeconomic difficulties.

## 6. Limitations

Several limitations of our study must be considered when interpreting the results. Firstly, the data utilized were extracted from the MIMIC-IV v3.1 database, which contains electronic health records from a single tertiary care center (Beth Israel Deaconess Medical Center). Therefore, the generalizability of our findings may be limited due to institution-specific practices, patient demographics, and clinical protocols, potentially differing from other healthcare settings.

Secondly, this retrospective cohort analysis relied on accurate documentation and coding practices. The use of ICD-9 and ICD-10 codes for patient selection may introduce potential misclassification bias, as coding practices can vary between providers and throughout time. Similarly, procedural interventions and PAD diagnoses could have been underreported or inconsistently recorded, leading to potential underestimations of PAD prevalence or procedure frequency.

Thirdly, the dataset lacked information on important variables such as disease severity (e.g., ABI scores and Rutherford or Fontaine classifications), smoking status, medication adherence, and outpatient follow-up data. The absence of these variables limits the study’s ability to fully account for confounding factors influencing PAD outcomes and procedural decisions.

Next, while in-hospital mortality was assessed, the absence of post-discharge follow-up data precludes the evaluation of long-term outcomes, such as limb salvage rates, functional status, quality of life, and long-term mortality. Consequently, the full impact of the observed sex disparities in procedural interventions on these critical outcomes remains unknown.

Finally, the observational nature of the study inherently limits the ability to establish causal relationships. Although sex disparities were evident, the underlying reasons—whether related to clinician biases, patient preferences, anatomical differences, or socioeconomic factors—could not be directly examined or conclusively established through this analysis.

## 7. Conclusions

This study provides compelling evidence that women with PAD face significant disparities in diagnosis, treatment, and intervention rates, despite experiencing comparable in-hospital mortality rates to men. The observed sex differences underscore the urgent need for improved clinical recognition, equitable access to vascular interventions, and targeted research efforts to address sex-based variations in PAD. By implementing sex-specific diagnostic and treatment strategies, we can move toward reducing healthcare disparities and optimizing PAD outcomes for women.

## 8. Future Directions

Future research should investigate whether anatomical differences influence procedural outcomes in women and whether sex-specific revascularization techniques may improve intervention success rates. Longitudinal studies examining post-procedural outcomes, limb salvage rates, and long-term mortality are needed to better understand the impact of sex-based differences in PAD management. Finally, prospective, sex-stratified clinical trials should be conducted to evaluate tailored medical and procedural interventions for women with PAD.

## Figures and Tables

**Table 1:** Summary statistics of the cohort.

Metric	Value
Total Patients	3913
Age (Mean ± SD)	69.70 ± 12.36
Age (Median, IQR)	70.0 (62.0–79.0)
Sex Distribution (%)	Male: 2326 (59.44)|Female: 1587 (40.56)
Race Distribution (%)	White: 2564 (65.53)|Black/African American: 406 (10.38)|Other: 943 (24.1)
Marital Status Distribution (%)	Married: 1692 (43.24)|Single: 967 (24.71)|Widowed: 737 (18.83) |Divorced: 353 (9.02)
Insurance Distribution (%)	Medicare: 2859 (73.06)|Private: 549 (14.03)|Medicaid: 426 (10.89)|Other: 64 (1.64)|No charge: 1 (0.03)
Hospital LOS (Mean ± SD)	11.03 ± 14.60
ICU LOS (Mean ± SD)	4.27 ± 6.10
In-Hospital Mortality Rate	6.80%
Patients Admitted to ICU	1223 (31.25%)
Procedure Counts	Amputation: 172|Atherectomy: 139|Endarterectomy: 332|Angioplasty: 562|Stent Placement: 577|Bypass Grafting: 629

**Table 2:** Sex breakdown in the cohort.

Metric	Female (F)	Male (M)
Total Patients	1587 (40.56%)	2326 (59.44%)
Mean Age	70.78	68.97
Median Age	72.0	69.0
ICU Admission Rate (%)	30.56	31.73
Mean ICU LOS (Days)	4.05	4.41
Median ICU LOS (Days)	2.0	2.0
Mean Hospital LOS (Days)	10.89	11.12
Median Hospital LOS (Days)	6.0	7.0
In-Hospital Mortality Rate (%)	6.62	6.92
Underwent Procedure Rate (%)	33.96	41.32
Marital Status Distribution	Widowed: 505|Married: 443|Single: 392|Divorced: 181	Married: 1249|Single: 575|Widowed: 232|Divorced: 172
Insurance Distribution	Medicare: 1197|Medicaid: 195|Private: 176|Other: 12|No charge: 1	Medicare: 1662|Private: 373| Medicaid: 231|Other: 52

**Table 3:** Procedural intervention rates by sex among PAD patients (% within sex group).

Sex	Amputation (%)	Atherectomy (%)	Endarterectomy (%)	Angioplasty (%)	Stent Placement (%)	Bypass Grafting (%)
Female	3.91	3.59	7.56	12.85	13.48	14.74
Male	4.73	3.53	9.11	15.39	15.61	16.98

**Table 4:** Key characteristics in patients over 60 years old summarized by sex.

Sex	Total Patients	Mean Age	ICU Admission Rate (%)	Procedure Rate (%)	Mean Hospital LOS (Days)	In-Hospital Mortality (%)
Female	1303	75.26	31.47	31.54	10.51	95 (7.29)
Male	1853	73.32	33.24	38.15	10.94	138 (7.45)

**Table 5:** Distribution of patients across different age groups, categorized by sex. Percentages represent the proportion of each age group within the respective sex category.

Age Group	Female (F)	Male (M)
<50	106 (10.1%)	139 (9.1%)
50–59	178 (16.9%)	334 (21.9%)
60–69	377 (35.8%)	713 (46.8%)
70–79	489 (46.5%)	677 (44.4%)
80–89	335 (31.8%)	383 (25.2%)
90+	102 (9.7%)	80 (5.3%)

## Data Availability

The data used for this retrospective cohort study can be found here: https://physionet.org/content/mimiciv/3.1/, accessed on 5 March 2025.

## Abbreviations

The following abbreviations are used in this manuscript:

PAD	Peripheral artery disease
MIMIC	Medical Information Mart for Intensive Care
